# Friction Stir Processing Influence on Microstructure, Mechanical, and Corrosion Behavior of Steels: A Review

**DOI:** 10.3390/ma14175023

**Published:** 2021-09-02

**Authors:** Neçar Merah, Mohammed Abdul Azeem, Hafiz M. Abubaker, Fadi Al-Badour, Jafar Albinmousa, Ahmad A. Sorour

**Affiliations:** 1 Mechanical Engineering Department, King Fahd University of Petroleum and Minerals, Dhahran 31261, Saudi Arabia; maazeem@kfupm.edu.sa (M.A.A.); abubaker982@gmail.com (H.M.A.); fbadour@kfupm.edu.sa (F.A.-B.); binmousa@kfupm.edu.sa (J.A.); sorour@kfupm.edu.sa (A.A.S.); 2 Interdisciplinary Research Center for Advanced Materials, King Fahd University of Petroleum and Minerals, Dhahran 31261, Saudi Arabia

**Keywords:** friction stir processing, steels, tensile, fracture toughness, fatigue, wear, corrosion

## Abstract

Friction stir processing (FSP) technology has received reasonable attention in the past two decades to process a wide range of materials such as aluminum, magnesium, titanium, steel, and superalloys. Due to its thermomechanical processing nature, FSP is used to alter grain structure and enhance mechanical and corrosion behavior in a wide range of steels. The refinement in grains and phase transformations achieved in steel after FSP affects hardness, tensile properties, fracture toughness, fatigue crack propagation rate, wear resistance, and corrosion resistance. A number of review papers are available on friction stir welding (FSW) or FSP of nonferrous alloys. In this article, a comprehensive literature review on the FSP/FSW of different types of steels is summarized. Specifically, the influence of friction stir processing parameters such as advancing speed, rotational speed, tool material, etc., on steels’ performance is discussed along with assessment methodologies and recommendations.

## 1. Introduction

Due to their excellent strength, fracture toughness, and high-temperature performance, steels are considered a crucial structural engineering material [1]. They are widely used in different industries such as automotive, aerospace, nuclear, chemical, oil and gas, military, construction, transportation, etc. Different categories of steel are used, depending on the application. Stainless steel is usually used in corrosive environments [2], while tool steels are used in high-temperature applications withstanding high wear, abrasion, and impact resistance. Carbon steels are relatively cheaper and widely used in applications not involving corrosive environments such as in buildings, rails, and machine tools. Lastly, alloy steels are a special class of steels tailor-made by adding specific amounts of alloying elements that modify steel properties and make them suitable for specialized applications [3]. Steels are mainly categorized based on their carbon composition, alloying elements, and production methods. Besides their composition, steel properties can be further modified using secondary processing techniques to alter the grain structure inside the material. It is well known that the response of a material to external loads, operating temperature, environment, etc., depends on its microstructure, i.e., the grain size/orientation and phase composition. There are many techniques by which the grain structure of steel can be altered or refined, such as normalizing, quenching, and annealing. These heat treatment techniques induce changes in the lattice or grain structure to improve specific properties such as tensile strength or hardness [4]. In addition to heat treatment processes, there are many well-established severe plastic deformation (SPD) techniques for grain refinement [5], such as equal-channel angular pressing [6], high-pressure torsion [7], multi-directional forging [8], and accumulative roll-bonding [9]. Friction stir processing (FSP) has recently been employed for grain refinement in steels [10]. Initially, friction stir processing/friction stir welding (FSP/FSW) were used on nonferrous alloys or low melting temperature alloys due to limitations of tool pin material. However, with the advent of new advanced materials designed for enormous toughness and high-temperature applications, FSP/FSW for steel or high melting temperature alloys was possible [11].

**Figure 1:**
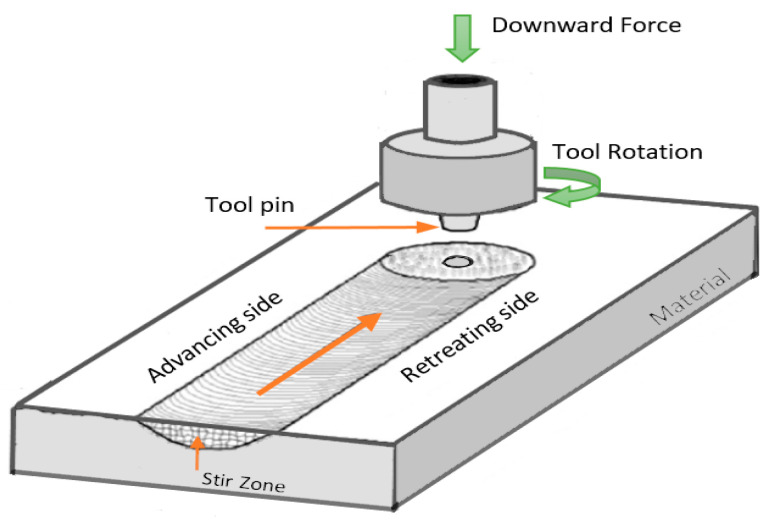
Schematic illustration of friction stir process.

Friction stir processing is an adaptation of the friction stir welding process, which is a thermomechanical joining technique [10]. FSW was developed at The Welding Institute (TWI) in 1991 and was used for joining difficult to weld metals and alloys. FSW is a solid-state technique wherein a non-consumable rotating tool with a specifically designed pin and shoulder is inserted between two metal surfaces. The tool rotates and traverses along the metal surfaces. The combined tool motion causes the softened materials (due to generated frictional and plastic heat by the tool rotation) to mechanically mix and create a joint [12]. The basic principle and procedure for FSW and FSP are the same, while the purpose is different. FSW is used for joining, while FSP is used for modification in the grain structure of the material. Friction stir processing (FSP) was developed by Mishra and Mahoney [13] to process a single piece of material. In FSP, a rotating tool mechanically stirs the material to alter its microstructure. The tool pin is introduced inside the material for processing, as illustrated in Figure 1. During this process, the tool pin penetrates the material and the shoulder of the tool embraces the surface of the material to make a close contact between tool and material. Severe plastic deformation occurs during FSP as the pin mechanically stirs the material to alter its microstructure. Grain refinement of the microstructure of the material occurs due to the plastic deformation and different researchers showed that it occurs through dynamic recrystallization of the material [13,14]. Ultrafine size grains can be formed by this process. As demonstrated in Figure 1, there are two sides in the processed material: the side of the workpiece, where the advancing direction and rotational speed direction of the tool are the same is termed as the advancing side (AS) and the other side, where they are opposite, is called as the retreating side (RS). The friction stir processing conditions/parameters, such as rotational speed, advancing speed, tilt angle, and plunging force, are critical for the microstructure and final properties of the processed material [15].

In addition to these processing parameters, the tool material and geometry play a crucial role in material flow and frictional heat in the processed zone. All these factors have an impact on the behavior of the material as they influence the grain structure [15]. By refining the grain microstructure, mechanical properties such as strength, hardness, fracture toughness, fatigue behavior, wear, and corrosion resistance can be altered [16,17,18]. In this article, a comprehensive review is undertaken to investigate the influence of FSP on the behavior of steels. Emphasis was given to research works addressing FSP as a surface modification technique for different types/grades of steels. Some papers on FSW were included mainly to discuss fracture toughness and fatigue behavior of steels due to lack of research in this area and also to cover a wide range of steels. A summary of various studies that are performed to find out the effect of FSP on different types of steels is categorized in terms of microstructural evolution, mechanical properties, and corrosion resistance. Critical outcomes are discussed in each section relating to the effect of FSP parameters on the performance of steel. Toward the end of this article, research methodologies adopted to characterize and assess the performance of steels after FSP is discussed, and finally, conclusions and recommendations to broaden the research area of FSP on steels are made.

## 2. Influence of FSP on Microstructure and Tensile Behavior of Steels

As mentioned earlier, FSP refines the grain structure of the material surface and results in a controllable phase transformation in the processed zone. The influence of this technique on the microstructure of the material has been studied by several researchers [19,20,21]. The influence of FSP on microstructure and tensile behavior of low carbon steel is presented in two separate studies [22,23]. Ultrafine grains with dual-phase structure were achieved using FSP under rapid water cooling for low carbon steel, giving it an enormous improvement in tensile and yield strengths while compromising its ductility. Ferritic grains with sizes ~1 μm were distributed around martensitic nanostructured (~200 nm) laths in friction stir processed (FSPed) samples. FSP was carried out using a pinless cermet tool at a rotation speed of 400 rpm and 50 mm/min travel speed [22]. Another FSP study on low carbon steel used in ship construction was performed at room temperature. A refined microstructure consisting of mainly ferrite (~3 μm), ferrite and cementite aggregates, and martensite was formed in the processed zone leading to enhancement in hardness and tensile strength with no variation in ductility. A tungsten carbide (WC) tool with a convex shoulder and a cylindrical pin was used in this investigation. FSP was performed at a rotation speed of 630 rpm and 45 mm/min traverse speed [23]. Similarly, Wang et al. [24] reported the formation of fine ferrite and cementite in friction stir processed high phosphorous mild steels SMA490AW and SPA-H, with a reduction of about eight times in average grain size as compared to the base material. The authors used the WC tool at a rotational speed of 80 rpm and a traveling speed of 150 mm/min. Recently, Anshari et al. [25] investigated the effect of heat input during FSP of medium carbon steel by varying tool traveling speed and fixing the rotational speed at 500 rpm. The authors studied the microstructural and mechanical properties of the stir zone (SZ). The authors reported the formation of three different phases within the SZ; hard phases martensite and bainite as well as soft phase of ferrite, at different percentages depending on process conditions. The traveling speed that resulted in enhanced plasticity was 80 mm/min leading to about 100% increase in both ultimate and yield strengths. Peng et al. [26] investigated the influence of FSP on AISI 316L steel produced by selective laser melting process. An improvement in hardness and tensile strength was reported due to refinement in grain size from 6.6 to 0.9 μm after FSP; furthermore, the dislocation density in the FSPed zone and grain orientation also contributed to the improvement in mechanical behavior. FSP was carried out using the WC tool with 37.5 mm/min and 375 rpm processing and rotation speed, respectively. Gotawala et al. [27] investigated FSP effects on the phase transformation in dual-phase DP600 steel, consisting of ferrite and martensite. The authors used a WC tool for FSP at a transverse speed of 40 mm/min and different rotation speeds of 1500, 1600, and 1700 rpm. This study showed the relevance of steel composition and initial phases present in BM in the phase transformation of FSPed steel. The high strength achieved due to the presence of martensite despite low carbon content in DP600 resulted in a non-diffusional transformation. The phase transformation consisted of ferrite, bainite, and martensite phases after FSP with finer bainite formed at a higher rotation speed. This transformation was found due to the high cooling rate from high temperatures generated during FSP with varying tool rotation speeds. Singh et al. [28] investigated the effect of FSP on microstructure, hardness, and tensile properties of SA210 grade A1 AISI steel. A pinless WC tool at 40 mm/min transverse speed and with 800 rpm and1400 rpm rotation speeds was used for two passes to evaluate the effect of FSP. The base metal ferritic and pearlite microstructure was transformed to fine-grained martensite and ferrite after FSP. Consequently, improvement in hardness and tensile strength was observed with an increase in rotation speed due to refinement in grain size and the presence of the martensitic phase.

A number of FSP/FSW studies have been carried out, particularly on duplex stainless steels (DSS), due to their advanced applications. Esmailzadeh et al. [29] investigated lean duplex stainless steel to find out the effect of various parameters of FSP on the microstructure of the material. FSP was carried out at a rotational speed of 800 rpm and at three different advancing speeds of 50, 100, and 150 mm/min using tungsten carbide FSWtool. Their findings revealed that the higher the advancing speed, the smaller the grain size in the processed zone, resulting in improvements in hardness and tensile strength of the material. The improvement in strength was attributed to more resistance to dislocation movements by an increase in the grain boundary density. Emami et al. [30,31] friction stir processed the duplex stainless steel SAF 2205 at a rotational speed of 400 rpm and an advancing speed of 50 mm/min using a tungsten carbide tool. Microstructural analysis showed that fine grains were produced in the processed zone due to high frictional heat and dynamic recrystallization. Similar findings were reported in other studies [32,33] for FSP of DSS wherein the FSP parameters were studied and refinement in microstructure was observed, and that the temperature and strain rate were found to be responsible for microstructural evolutions in the stir and thermomechanical-affected zones. A custom-made FSP tool from the WC-Co rod was used to investigate rotational speeds in the range of 800–1600 rpm and traverse speeds in the range of 12 to 40 mm/min, whereas in other studies, the W-Re tool was used at 300 rpm and 100 mm/min travel speed. The extent of refinement from the strain, strain rate, and temperature during FSP was found to be dependent on the ratio of traverse speed and the rotational speed in the case of the WC-Co tool. Saeid et al. [34] investigated the same SAF 2205 DSS to examine the effect of different parameters of FSW on the microstructure and tensile behavior. FSW was executed at a rotational speed of 600 rpm and varying advancing speeds of 50–250 mm/min using a tungsten carbide stir tool. It was found that by increasing the advancing speed from 50 mm/min to 200 mm/min, grain structure became more refined in the processed zone; however, a groove-like defect was produced at 250 mm/min. This was due to the fact that frictional heat is insufficient to soften the material around the spinning tool at this higher welding speed, resulting in a lack of material flow, consequently creating fill defects in the processed zone. The authors concluded that due to the reduction in grain size, hardness, and tensile strength of the DSS were increased. It was also reported that the grain sizes in the stir zone were not of the same size, and there was more grain refinement on the advancing side compared with the retreating side of the processed zone indicating the asymmetric grain structure in FSPed zone [35]. Santos et al. [36] studied the effect of FSW on four duplex stainless steels UNS S32750, S32205, S32101, and S32760, with the tool traverse speeds of 50–150 mm/min and the rotational speeds of 200–600 rpm using polycrystalline cubic boron nitride (PCBN) stir tool. They noticed that the advancing side region experienced higher strains than the retreating side, resulting in more grain refinement. It was also observed that the stir zone hardness was higher in comparison with the base metal. The microstructure refinement increased the grain boundary density thereby showing increased yield and tensile strengths in all DSS grades.

**Figure 2:**
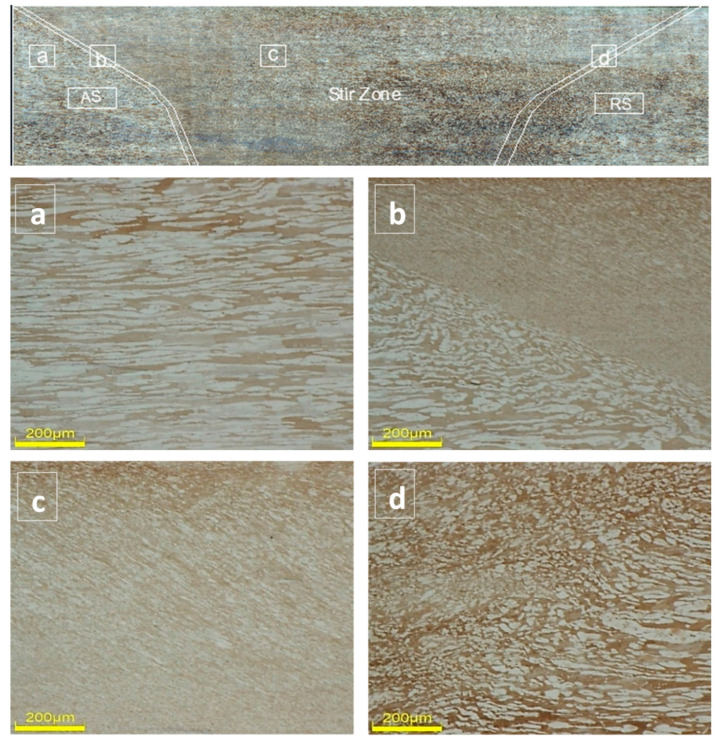
Typical microstructures of friction stir processed material: (**a**) base metal, (**b**) boundary of base metal and stir zone on advancing side, (**c**) stir zone, (**d**) boundary of base metal and stir zone on retreating side [16].

Using a lanthanide tungsten (W-1La_2_O_3_) tool, Mishra et al. [37] studied FSW of 2507 super duplex stainless steel (SDSS) to find out the effect of varying advancing speed (10–175 mm/min) on the microstructure of this material considering the constant rotational speed of 800 rpm. It was observed that with the increase in advancing speed, grain size reduced until 100 mm/min then increased again. Similarly, Ma et al. [38] investigated 2507 DSS using the W-25Re FSP tool with a transverse speed in the range of 50–200 mm/min and a constant rotation speed of 400 rpm. The dominating factor for grain size refinement in FSP at transverse speed from 50 to 100 mm/min was attributed to a decrease in heat input that restricted grain growth, whereas speeds ranging from 100 to 200 mm/min showed a gradual increase in grain size due to lower strain rate. No significant effect in the phase composition was found for this type of steel with an increase in transverse speed due to lower heat input. Furthermore, the tensile strength of FSPed sample was improved from 873 to 1083 MPa accompanied by a decrease in elongation from 45% to 20.8% at 100 mm/min transverse speed. Both studies reported the heat input and strain rate as crucial factors determining grain size refinement in the FSPed zone. In a separate work by Sato et. al. [39] on FSW of the same 2507 SDSS material performed using the PCBN tool at a rotational speed of 450 rpm and 60 mm/min traverse speed, the FSW developed a sharp boundary on the advancing side of the processed zone and on the retreating side, there was a gradual decrease in grain size from the base material (BM) to the stir zone. Similar findings of an increase in strength and hardness by grain refinement of ferrite and austenite phases were reported. Figure 2 shows the grain structure of the base and friction stir processed 2507 SDSS. It can be observed from Figure 2 that a large reduction in grain size has been achieved from 160 μm in the base metal (Figure 2a) to 30 μm in the processed zone (Figure 2c). In addition, it can be seen that the grain size varies in the stir zone from the advancing side (Figure 2b) to the retreating side (Figure 2d), showing the heterogeneity of the grain structure in the processed region [16]. Table 1 summarizes the findings of different studies on the tensile behavior of steel after FSP/FSW.

From the above-mentioned studies, it can be concluded that FSP/FSW refines the grains, thus creating smaller size grains with an increase in the grain boundary density. In addition, grain structure in the processed material was found to be asymmetric, with higher grain refinement on the advancing side compared with the grains on the retreating side. The tensile and hardness behavior of the material improved because larger number of grain boundaries are obstructing the dislocation movement in the processed material; however, it may reduce the elongation and ductility by making the processed material more brittle.

## 3. Effect of FSP on Fracture Toughness and Fatigue Behavior

Fracture toughness describes the ability of the material to resist crack growth. Materials having higher fracture toughness resist the crack growth more than the material of lower fracture toughness [40]. As discussed above, FSP refines the grain structure, so it is expected to alter the fracture toughness of the processed material. Different researchers have examined the effect of FSP/FSW on the fracture toughness of different grades of steel and stainless steel material [16,41,42,43,44,45]. However, fracture toughness dependence on FSP parameters is still not very clear. Different studies have been performed on different materials to find out a proper relationship between fracture toughness and FSP parameters, but their findings were different. Some stated reduction in fracture toughness by FSP, and some reported that fracture toughness of BM and friction stir processed material are comparable, and some revealed increase in fracture toughness by FSP. However, there is an agreement in the literature that the change in fracture toughness by FSW/FSP depends on welding/processing parameters, tool material, and the base material. Meinhardt et al. [41] investigated the influence of FSW on the fracture toughness of UNS S32760 SDSS. They have found a 19% decrease in fracture toughness within the friction stir welded (FSWed) zone. Fairchild et al. [42] investigated friction stir welded pipeline steel, grades X65 to X120. These welds were made at 300 rpm rotational speed and 51 mm/min advancing speed using a PCBN FSW tool. Their results showed that fracture toughness of weld material was significantly lower compared with BM. Similar work was performed by Santos et al. [43] on ISO 3183 X80M steel, revealing that by using higher spindle speed (around 500 rpm), the fracture toughness of weld metal was reduced, but for lower spindle speeds (around 300 rpm), fracture toughness of the weld metal was higher compared with BM. Similar results were stated by Tribe and Nelson [44] for API X80 steel, who also found that fracture toughness was dependent on heat input and rotational speed. They also showed that due to microstructure heterogeneity in the stir zone, the fracture toughness was different in the processed zone from weld centerline to either direction. Avila et al. [45] investigated the pipeline steel API-5L-X80 to examine the behavior of fracture toughness of friction stir welded joints as compared with BM. The welding parameters used for FSW were 300 rpm and 100 mm/min of rotational and advancing speeds, respectively. It was revealed that the stir zone produced by two passes of FSW had higher fracture toughness values than that of the stir zone of single-pass weld samples, which in turn was superior to that of the BM. The influence of FSP on fracture toughness of 2507 SDSS [17] was evaluated using cubic boron nitride and 30% W-Re tool at 400 rpm and 100 mm/min speed. The fracture toughness was evaluated on compact tension specimen according to the J-integral approach of ASTM standard E1820-20. Enhancement in fracture toughness was reported for samples before and after corrosion due to FSP. It is worth mentioning that fracture toughness of submerged arc welded super duplex stainless steel SAF 2906 [46] without FSP was not comparable to FSPed 2507 SDSS. Table 2 summarizes the findings of different studies on fracture toughness behavior of the steels after FSP/FSW. It can be concluded that the fracture toughness depends on FSP parameters as well as on workpiece and tool materials. However, there is no specific trend on how fracture toughness evolves with each of these variables. Table 2 summarizes some of these findings.

For the same reasons illustrated above, FSW/FSP also has an influence on the fatigue behavior of the material, and there are various studies available in the literature that describe the effect of FSP on the fatigue behavior of nonferrous alloys, mainly aluminum-based, but steels did not receive the same attention. For instance, the fatigue behavior of FSWed DH36 marine grade steel was carried out, and new S-N curve parameters were recommended for low alloy FSWed steels. The fatigue resistance of FSW butt joints was found to be much higher than other classes of welded butt joints. It was also recommended that slow FSW speed is best suited for improved fatigue performance due to the development of a highly refined, defect-free microstructure in the welded zone [47]. Fatigue strength of butt welded joints after FSP was investigated by Yamamoto and Ito [48] on two high-strength steels. Double-sided FSP was performed using a WC-6% Co tool with a rotational speed of 400 rpm and travel speed of 140 mm/min. The FSPed specimens were tested under tensile fatigue, performed at a stress ratio of 0.1 and 20 Hz frequency. An improvement in fatigue strength was observed for steel grades at 10^7^ cycles as a fatigue limit. Fatigue behavior of low carbon DC04 interstitial free steel was also investigated, and fatigue life was found to be enhanced for FSPed steel both in air and corrosive NaCl environments. FSP was performed on two sides of a steel plate using a cylindrical WC tool with a traverse speed of 60 mm/min and a rotation rate of 950 rpm. Fatigue tests were conducted at a loading frequency of 10 Hz and a stress ratio of 0.1, both in air and 3.5 wt% NaCl solution. The fracture surface of FSPed steel depicted lower fatigue crack growth rate, and enhancement in fatigue limit was found from 200 to 400 MPa at 10^7^ cycles for samples tested in the air [49]. Similar findings in fatigue behavior of tool steel DP600 were reported wherein the FSP improved fatigue limit from 350 to 480 MPa at 10^6^ stress cycles [50]. Recently, the fatigue behavior of SAF-2507 SDSS was investigated by Arafat et al. [18], using fatigue crack propagation (FCP) tests on the CT samples at three different stress levels of 66, 94, and 113 MPa. The FCP tests were conducted at a constant load amplitude with a stress ratio of 0.1 at a frequency of 10 Hz. The authors found that friction stir processing improved both crack initiation and propagation lives of SAF 2507.

From the above-mentioned studies, it can be stated that FSP increases the fatigue life and strength of steel materials. This improvement in fatigue behavior of the material by FSP is again due to the refinement of the microstructure of the material and elimination of defects. As in refined grain structures, the increased strength according to the Hall-Petch relationship improves the nucleation resistance of fatigue cracks, and propagation becomes more difficult, resulting in an enhancement in the fatigue life of the material [18].

## 4. Effect of FSP on Corrosion Behavior of Steels

There are many studies, which showed severe negative effects of corrosion on the performance of steels [51,52]. As discussed in earlier sections, the modification of steel surface by friction stir processing has shown enhancement in the mechanical behavior of steel; however, the effect of corrosion on these properties and the rate of corrosion is critical for the application of steels in corrosive environments. Sarlak et al. [53] examined the influence of FSW on the corrosion behavior of lean DSS. They performed FSW at 800 rpm rotational speed and 50, 100, and 150 mm/min advancing speeds. The welded material was immersed in an H_2_SO_4_ solution, and corrosion resistance was evaluated by potentiodynamic polarization (PDP). The stir zone showed better corrosion resistance because of grain refinement. The corrosion resistance was further enhanced by raising the advancing speed from 50 to 150 mm/min. Ajith et al. [54] investigated friction-welded UNS S32205 DSS and found that friction-welded specimens showed superior corrosion resistance than base metal in 3.5% NaCl solution. Similar research was presented on 2507 SDSS FSPed at 50 mm/min of advancing speed and 600 rpm of rotational speed by Mishra et al. [55]. The corrosion resistance was assessed by PDP, open-circuit potential (OCP), and electrochemical impedance spectroscopy (EIS), which all indicated better corrosion behavior in FSPed samples compared with BM. Recent work on AISI 440C high carbon martensitic stainless steel examined the influence of FSP on corrosion resistance of the material [56]. FSP was executed at a rotational speed of 2000 rpm and processing speeds ranging from 150 to 300 mm/min, using W-Re tool. PDP tests were performed in 3.5% NaCl solution, and results showed an increase in corrosion resistance for the FSPed sample processed at 150 mm/min due to the formation of a thick passive film. Huang et al. [57] investigated the corrosion behavior of low carbon steel after FSP was performed in two separate environments, air and water. Phase transformation and dynamic recrystallization observed during FSP resulted in fine-grained ferrite and martensite in the processed zone. A more uniform distribution of fine martensite lath was formed when FSPed underwater. PDP and EIS tests were conducted in 3.5 wt% NaCl solution at room temperature. Due to the presence of dense and uniform passive film, FSPed steel specimens underwater showed better electrochemical corrosion resistance. AISI D2 tool steel was also investigated for corrosion behavior after FSP [58]. The formation of fine carbides in the ferritic-martensitic matrix was observed due to FSP, as seen in other studies for the same steel grade. FSP was carried out using the WC-Co tool at 385 mm/min traverse speed and 400–800 rpm rotational speed. Corrosion behavior was assessed in 3.5 wt% NaCl solution using the PDP test, corrosion potential, and current density through the Tafel plot method. The corrosion resistance of FSPed tool steel was found to be enhanced due to a high fraction of low-angle grain boundaries except for samples processed at 800 rpm due to less homogenous distribution of carbide particles. Another steel type 2507 SDSS was also analyzed for corrosion behavior [17] after FSP using PDP and linear polarization resistance (LPR) tests in 3.5 wt% NaCl medium. The results of these tests showed improvement in corrosion resistance of FSPed SDSS by about 86%, owing to the presence of an oxide layer containing Cr, which is formed by an increase in grain boundary density in the FSPed zone. For pipeline applications, Giarola et al. [59] studied the corrosion performance of friction stir welded API X70 steel using microcell and PDP to locally investigate the corrosion behavior of SZ, thermo-mechanically affected zone (TMAZ), heat-affected zone (HAZ), and BM in 3.5% NaCl. The authors used a composite tool of PCBN-WRe at a traveling speed of 100 mm/min and a rotational speed of 300 rpm. The minimum corrosion rate was found at the SZ, three times less than that of BM. Table 3 summarizes the findings of different studies on corrosion resistance behavior of different types of steels after FSP/FSW depicting enhancement in corrosion resistance.

## 5. Effect of FSP on Wear Behavior of Steels

The wear resistance is affected by the material’s hardness and toughness. As FSP affects the grain size and, consequently, hardness and toughness, it will also affect the wear properties. Methods such as nitriding and carburizing have been used to increase surface hardness and wear resistance in steels. The rapid surface hardening along with localized grain refinement achieved by FSP of steels is beneficial for improving wear behavior and other properties. Aldajah et al. [60] studied high carbon steel (AISI 1080) to examine the influence of FSP on its wear behavior. Wear testing was performed with a pin-on-disk tribometer using an alumina ball with 10 N force and 0.05 m/s speed for 1 h. The wear rate was determined to be 87% less in the FSPed sample. Similar results were reported for low carbon steel [61], and enhancement in wear resistance was achieved due to an increase in hardness and strength after FSP. However, at a higher applied load of 15N in the wear test, the decrease in wear rate was not significant. Tinubu et al. [62] investigated the effect of FSP on the wear performance of Fe-Ni-Cr-based austenitic precipitation hardened A-286 stainless steel alloy. They determined that along with an increase in microhardness, the wear rate in the stir zone was reduced from 1.0× 10^−6^ to 5.8 × 10^−7^ mm^3^/Nm due to FSP. The increased microhardness and improved wear resistance were attributed to the combined effects of FSP-induced microscopic grain refinement and finer-scale microabrasion in the FSPed zone. Similar findings were reported by Eskandari et al. [63] in FSP of AISI 430 ferritic stainless steel wherein mild adhesive wear with 17.4 times improvement in wear resistance. The formation of the dual-phase microstructure of ferrite and martensite with fine grains on the surface was found responsible for improved hardness and wear resistance in this type of steel. Another variant AISI 420 martensitic stainless steel [64] was FSPed and investigated for tribological performance under the dry reciprocating ball on plate test and micro-scale abrasive wear test. The FSPed AISI 420 stainless steel reached a maximum hardness of 722 HV and improved wear resistance compared to similar heat-treated steels such as D2 tool steel. Yasavol and Ramalho [65] investigated the effect of tool rotation speed on wear properties of FSPed AISI D2 tool steel. FSP of this steel revealed a homogenous distribution of carbide particles in a refined stir zone. Another important aspect of the FSP effect on this type of steel was investigated, which had to do with the grain orientation and resultant textures developed during FSP at various tool rotation speeds. Typically, FSPed steel with tool rotational speed of 500 rpm was found to have the highest nano-hardness (1100 HV) despite lower grain refinement than 800 rpm tool rotational speed. Using electron backscatter diffraction (EBSD) analysis of stir zone, the FSP at 500 rpm tool rotation speed resulted in the arrangement of grains with a high density of loose-packed planes. Moreover, the lowest specific wear rate of 0.32 × 10^−14^ m^2^/N was found at a tool rotational speed of 500 rpm. Another tool steel grade SKD61, was friction stir processed using the PCBN tool at a rotational speed of 300 rpm and travel speed of 30 mm/min. Reciprocating friction testing machine under the dry sliding condition against alumina ball was used to evaluate the friction and wear characteristics of the surface of the friction stir processed specimens. The wear test was carried out at a fixed distance of 5 mm and vibration frequency of 2 Hz for 2 h under a load of 1.96 N. The results of wear tests showed a decrease in wear width and depth by 62% and 86%, respectively for the FSPed zone in tool steel material, owing to a substantial increase in microhardness from 200 to 800 HV in the stir zone [66]. The microstructure and consequently the wear rate of FSPed steel was found to be dependent on the design of the tool pin. The square pin profile was found to be the best for grain refinement due to the intensive plastic deformation in the processed zone [67]. Lorenzo-Martin and Ajayi [68] used FSP as surface treatment for AISI 4140 alloy steel and studied its tribological performance. The results revealed FSP as a favorable process to enhance surface hardness of sample and improve wear resistance by two folds compared to conventional heat treatment processes for this type of steel.

For improving tribological properties of low carbon steel, Mostafa et al. [69] used multipass FSP to produce steel/silicon carbide (SiC) nanocomposite. The authors investigated the impact of tool rotational speed and traveling speed on hardness as well as wear rate, and it was found that after four passes, the hardness of SZ was three times larger than that of base metal. The increase in the number of passes resulted in the dispersion of the nanosized SiC, forming a homogenous distribution. In addition, SiC resulted in reducing the wear rate three times as compared to friction stir processed sample. In another study, titanium powder was mixed in low carbon steel (St37) with the aid of FSP and further treated with plasma nitriding to form low carbon steel/titanium nitride surface composite [70]. This method of using FSP coupled with plasma nitriding to prepare surface composites resulted in an enormous increase in surface hardness from 130 to 823 HV, due to the formation of the nitride layer on the surface. The wear resistance was improved by 43.8% after FSP and further improved after plasma treatment. FSP was carried out at a tool rotation speed of 800 rpm and 36 mm/min advancing speed using the WC-Co FSP tool. Holes were drilled on the surface of BM to incorporate titanium powder during FSP. Studies related to steel’s wear or erosion resistance in hydraulic environments, due to cavitation were also investigated. FSP of 304/304L austenitic stainless steel was conducted using PCBN with 30 wt%W-Re tool at 50 rpm and 25.4 mm/min traverse speed. Cavitation erosion testing by liquid jet was performed conforming to ASTM G134 on specimens in a closed chamber. The cavitation erosion resistance of the FSPed samples was enhanced by a factor of 3.3 owing to grain refinement and a 50% increment in hardness at a relatively lower processing temperature of 740 °C [71]. FSP of hydro turbine 13Cr4Ni steel also showed 2.4 times higher resistance against cavitation erosion compared to the base metal. Escobar et al. [72] investigated the influence of FSP on cavitation erosion resistance of duplex stainless steel UNS S32205. FSP was performed, using the PCBN-40%W-Re tool at 200 rpm and 100 mm/min speed. A cavitation erosion test was performed in ultrasonic equipment filled with water, and the specimen is kept underneath a vibrating horn at a 0.5 mm distance. FSPed samples were treated for 16 h before measuring the weight loss. The results showed improvement in cavitation erosion resistance of FSPed DSS specimens due to an increase in hardness, grain refinement, and most importantly, due to the smaller equiaxed ferritic and austenitic grains, which shortens the length of the ferrite and austenite interface. Therefore, these microstructure modifications hinder crack propagation and dislocation movement, resulting in a reduced erosion rate. Table 4 summarizes the findings of different studies on the wear behavior of different types of steels after FSP/FSW depicting enhancement in wear resistance.

## 6. Research Methodologies

The research methods used to investigate the effect of FSP on the behavior of a material are generally experimentally based. These methods include friction stir processing of the material under specific processing parameters so as to achieve a smooth defect-free processed zone with grain refinement and then carry out characterization/tests to study the behavior of the processed material. Different researchers used different FSP parameters (advancing speed, rotational speed, tilt angle, and tool material and geometry) relative to the nature of the material being processed and machine limitations. The samples from the processed zone are machined/prepared as per the standards required for mechanical and corrosion tests.

Initial studies on FSP of steels targeted weld joints and plates from carbon steels and tools steels to enhance their microstructure and tensile behavior. Microstructure studies on FSPed specimens were generally conducted on the cross-section perpendicular to the FSP/FSW direction using optical microscopy (OM) and EBSD technique to evaluate grain size, grain orientation, and phase composition. Scanning electron microscopy (SEM) equipped with energy dispersive spectroscopy (EDS) is used to analyze the microstructure and elemental composition in specimens along with X-ray diffraction to identify and quantify phases. Vickers microhardness test is commonly used to evaluate the distribution of hardness in the processed region. Tensile testing is carried out following the ASTM E8 standard, where the tensile specimens are machined from the processed zone along the processing direction. So various researchers used a similar methodology to investigate the microstructure of the processed region and to examine the hardness and tensile behavior of the processed sample.

The fracture toughness of FSPed specimens is generally analyzed using the crack tip opening displacement, J-Integral and stress intensity factor methods with single-edge notch bend and CT samples according to ASTM E 1820 standard. Fatigue testing is usually performed on servo-hydraulic systems using a positive load ratio and frequencies ranging from 10–80 Hz. So various researchers used similar methodology (but different testing parameters) to investigate the fracture and fatigue behavior of the processed and base metal samples.

The corrosion behavior of the FSPed and BM is usually performed under controlled lab-scale experiments using corrosive mediuma such as H_2_SO_4_ or NaCl solutions, and corrosion resistance is usually evaluated by potentiodynamic polarization, open-circuit potential, electrochemical impedance spectroscopy, or linear polarization resistance methods. Lastly, the wear testing is carried out using tribometers involving pin-on-ring or pin-on-disk wear tester with different applied loads and sliding velocities against relatively hard and tough disk material. Wear resistance is determined by the weight loss method. Wear testing is also performed on a reciprocating wear tester against a cylindrical hard surface with a definite load and sliding speed. The cavitation erosion test was performed in a closed environment filled with water according to ASTM G32 standard for ultrasonic treatment, whereas other methods such as liquid jet impingement are also used as per ASTM G134 to evaluate cavitation erosion rate. Table 5 summarizes the types of studies conducted on various types and grades of steel.

The reliability and validity of the results from the literature review of FSP/FSW of steels are found to be relevant or acceptable in most cases. Since FSP is performed experimentally, there are several parameters that could drastically affect the final results. In addition, FSP is critically dependent on the tool and machine parameters (robust and precise machining is required for FSP; it is very difficult for researchers to maintain exactly the same conditions during experiments when dealing with different materials). Moreover, the frictional heat developed along with severe mechanical mixing in the processing zone develops heterogeneous microstructure, which could lead to the difference in properties of processed materials. However, comparing the literature of FSP/FSW on different types of steels has shown similarity with respect to microstructure refinement. Many studies have shown relevance to FSP parameters on the final grain structure and phase compositions in steels. The findings of improvement in tensile properties and hardness for different grades of steel are comparable. This improvement was mainly due to an increase in grain density, i.e., grain boundary strengthening achieved in the FSPed zone. The refined microstructure with a higher number of grain boundaries in the processed zone also resisted the crack growth more compared with the lower number of grain boundaries. So, fatigue crack propagation resistance and fracture toughness improved with the increase in the density of grain boundaries in the processed region. Similarly, the wear and corrosion resistance of friction stir processed steels were improved.

## 7. Conclusions and Recommendations

Based on the above literature review, it can be concluded that:

- FSP refines the grain structure in all types of steels. So, FSP is expected to, in general, affect their mechanical and corrosion behavior.
- The grain structure of the processed material is asymmetric; grains on the advancing side of the processed material are more refined than those on the retreating side with different orientations/textures.
- FSP increases tensile strength and hardness. However, it reduces the ductility in most types of steels.
- Fracture toughness may be increased or decreased by FSP, depending on FSP parameters, tool geometry, and steel grade.
- FSP enhances the fatigue life of the processed material and increases the fatigue strength of the FSWed joints in steel. The results obtained on SDSS show excellent improvement in both fatigue initiation and propagation lives.
- FSP improves the corrosion resistance in steels due to changes in surface compositions and the formation of stable passive films. It also enhances the wear resistance in all types of steels due mainly to the hardened FSPed surface.
- The properties of the processed material are found to be highly dependent on the advancing and rotational speeds of the FSP tool. There is no obvious correlation between these parameters and the behavior of the processed material. So, an intensive investigation is required to assess the influence of these parameters on the performance of processed steels.
- Various studies showed different percentage improvements in the specific properties of the investigated steels. So, it means that the parameters of FSP such as the advancing speed, rotational speed, tilt angle, and tool material/geometry play an important role in the final behavior of steels. The influence of each parameter of FSP on the properties of steel needs to be investigated thoroughly.

## Figures and Tables

**Table 1:** Effects of FSP/FSW on tensile behavior and hardness on different types of steel.

Material	FSP/FSW Parameters	Tool Material	Findings	Ref.
SAF 2507	FSP at 400 RPM 100 mm/min	PCBN	14% improvement in tensile strength and 11% in hardness.	[16]
Lean DSS	FSW at 800 RPM 50–150 mm/min	WC-based	3.5–11% Increase in tensile strength. Increase in hardness.	[29]
lean DSS	FSP at 300 RPM 100 mm/min	W-Re	4% decrease in tensile strength. 2% decrease in elongation.	[32]
2205 DSS	FSW at 600 RPM 50–250 mm/min	WC-based	Increase in tensile strength. Increase in hardness.	[34]
2507 SDSS	FSP at 800 RPM 10–175 mm/min	Lanthanide tungsten	7% increase in tensile strength. 28% decrease in ductility.	[37]
2507 SDSS	FSW at 450 RPM 60 mm/min	PCBN	Tensile strength was comparable. 40% decrease in elongation. Increase in hardness.	[39]

**Table 2:** Effects of FSP/FSW on fracture toughness.

Material	FSP/FSW Pfarameters	Tool Material	Findings	Ref.
SAF 2507 SDSS	FSP at 400 RPM 100 mm/min	PCBN	12% improvement in fracture toughness.	[16]
S32760 SDSS	FSW at 400 RPM 180 mm/min	PCBN	19% reduction in fracture toughness in welded area.	[41]
Pipeline Steel	FSW at 300 RPM 51 mm/min	PCBN	Significantly lower fracture toughness in welded area.	[42]
3183 X80M-Steel	FSW at 300–500 RPM 100 mm/min	PCBN	Fracture toughness depended on the rotational speed.	[43]
API X80 Steel	FSW at 303–725 RPM 82–287 mm/min	PCBN	Fracture toughness depended on the rotational and advancing speed.	[44]
API-5L- X80 Steel	FSW at 300 RPM 100 mm/min	PCBN W-Re	Significantly lower fracture toughness for single-pass weld and comparable fracture toughness for two-pass weld.	[45]

**Table 3:** Influence of FSP/FSW on corrosion resistance.

Material	FSP/FSW Parameters	Medium for Corrosion Test	Findings	Ref.
2507 SDSS	FSP at 400 RPM 100 mm/min	3.5 wt% NaCl	86% improvement in corrosion resistance.	[17]
Lean DSS	FSW at 800 RPM 50–150 mm/min	H_2_SO_4_ solution	33–53% increase in corrosion resistance.	[53]
2507 SDSS	FSP at 600 RPM 50 mm/min	3.5 wt% NaCl solution	63–69% increase in corrosion resistance.	[55]
AISI 440C	FSW at 2000 RPM 150, 200, and 300 mm/min	3.5 wt% NaCl solution	10 times reduction in corrosion current density and improved pitting resistance.	[56]
AISI D2 Tool Steel	FSW at 400–800 RPM 385 mm/min underwater	3.5 wt% NaCl solution	No improvement or degradation in corrosion resistance.	[58]

**Table 4:** Influence of FSP/FSW on wear resistance.

Material	FSP/FSW Parameters	Wear Test/Parameters	Findings	Ref.
2507 SDSS	FSP at 400 RPM 100 mm/min	Ball-on-disk 25–100 N, 0.1 m/s	15–26% improvement in wear resistance	[17]
AISI 1080	FSP at 1000 RPM 15 mm/s	Pin-on-disk 10 N, 0.05 m/s	~87% improvement in wear resistance	[60]
A-286 SS	FSP at 400 RPM 25 mm/min	Reciprocating ball on plate 0.2 N, 20 Hz	~42% improvement in wear resistance	[62]
AISI 430 SS	FSP at 1400 RPM 16 mm/min	Pin-on-disk 30 N, 0.1 m/s	~94% improvement in wear resistance	[63]
AISI D2 tool steel	FSP at 385 mm/min 400–800 RPM	Reciprocating ball on plate 10 N, 4.5 Hz	Wear resistance improved at 500 RPM	[65]

**Table 5:** Experimental studies available in the literature investigating the influence of FSP on the behavior of different types/grades of steels.

Type of Study	Analysis Technique/Testing Standards	Steel Type/Grade [References]
Microstructure Study	Optical microscopy Scanning electron microscopy, Energy dispersive Spectroscopy X-ray diffraction Electron back scatter Diffraction ASTM E3 -01 ASTM E112-12 ASTM E1806-18	2507 SDSS [16,37,39]
		Low carbon steel [22,23,57]
		Lean DSS [29,32,34,35]
		2205 DSS [30,31,33]
		DP600 [27]
		S32760 SDSS [41]
		API 5L X80 [43,44,45]
		DH36 [47]
		HSS [48]
		AISI 440C [56]
		AISI D2 [58]
		High carbon steel [60]
		A-286 SS [62]
		AISI 430, 420 [63,64]
		304/304L SS [71]
		S32205 DSS [72]
Hardness and Tensile properties	Vickers microhardness/ASTM E92 Universal testing machine/ ASTM E8M-04	2507 SDSS [16,37,39]
		Low carbon steel [22,23,57]
		Lean DSS [29,32,34,35]
		2205 DSS [33]
		DH36 [47]
		AISI D2 [58]
Fracture Investigation	Crack tip opening displacement single-edge notched bend bar Compact tension specimen ASTM E 1820 ASTM E 399	2507 SDSS [17]
		S32760 SDSS [41]
		API 5L X80 [43,44,45]
		HSS [48]
Fatigue Behavior	Compact tension specimens Servo-hydraulic system Bending fatigue system Residual stress ASTM E466 ASTM E647	2507 SDSS [18]
		DH36 [47]
		Low carbon steel [49]
		DP600 [50]
Corrosion Study	PDP, OCP EIS, LPR ASTM G31-72	2507 SDSS [17,55]
		Low carbon steel [49,57]
		Lean DSS [53]
		AISI 440C [56]
		AISI D2 [58]
		API X70 [59]
Wear Examination	Pin-on-ring or pin-on-disk/ASTM G99 -17 Reciprocating ball on plate/ ASTM G133 -05 Abrasive wear test/ ASTM G195 -18 Cavitation erosion test ASTM G32, ASTM G134	2507 SDSS [17]
		High carbon steel [60]
		Low carbon steel [61]A-286 SS [62]
		AISI 430, 420 [63,64]
		AISI D2 [65]
		AISI 4140 [68]304/304L SS [71]
		S32205 DSS [72]

## Data Availability

This is a review paper, all reported data is taken from the mentioned references.
